# Revisiting the Role of Potassium Sensitivity Testing and Cystoscopic Hydrodistention for the Diagnosis of Interstitial Cystitis

**DOI:** 10.1371/journal.pone.0151692

**Published:** 2016-03-21

**Authors:** Yuan-Hong Jiang, Jia-Fong Jhang, Hann-Chorng Kuo

**Affiliations:** Department of Urology, Buddhist Tzu Chi General Hospital and Tzu Chi University, Hualien, Taiwan; Cedars-Sinai Medical Center, UNITED STATES

## Abstract

**Objectives:**

To revisit the diagnostic roles of cystoscopic hydrodistention and the potassium sensitivity test (PST) for the diagnosis of interstitial cystitis (IC).

**Methods:**

We prospectively enrolled 214 patients clinically diagnosed with IC, 125 non-IC patients who underwent video urodynamic studies and PST, and another 144 non-IC patients who underwent cystoscopic hydrodistention before transurethral surgery. The sensitivity, specificity, and positive and negative predictive values were calculated for the PST and glomerulations after cystoscopic hydrodistention.

**Results:**

After cystoscopic hydrodistention, glomerulations developed in 211/214 (98.6%) IC patients and 61/144 (42.4%) of the non-IC patients including patients with stones (45/67, 67%), hematuria (2/5, 40%), and stress urinary incontinence (SUI) (6/17, 35%). When positive glomerulation was defined as grade 2 or more, the sensitivity was 61.7%. The PST was positive in 183/214 (85.5%) IC patients and 7/17 (41%) with hypersensitive bladder, 7/32 (22%) with detrusor overactivity, 5/27 (18%) with SUI, 2/21 (10%) with lower urinary tract symptoms, and 2/25 (8%) with bladder outlet obstruction. The PST had a sensitivity of 85.5% and a specificity of 81.6% for diagnosis of IC. IC patients with a positive PST had a significantly smaller urgency sensation capacity, smaller voided volume, and greater bladder pain score.

**Conclusions:**

Both the PST and glomerulations after hydrodistention are sensitive indicators of IC, but the specificity of glomerulations in the diagnosis of IC is lower than that of the PST. A positive PST is associated with a more hypersensitive bladder and bladder pain, but not the grade of glomerulations in IC patients. Neither test provided 100% diagnostic accuracy for IC, we might select patients into different subgroups based on different PST and hydrodistention results, not for making a diagnosis of IC but for guidance of different treatments.

## Introduction

Interstitial cystitis (IC) is characterized by bladder pain associated with urgency, frequency, nocturia, and sterile urine [1]. The diagnosis of IC is based on the symptomatology and urological findings including characteristic cystoscopic features after hydrodistention under anesthesia [2]. Although the disease has been known for more than a century, the pathophysiology of IC remains unclear, and its diagnosis is based on the exclusion of other diseases [3].

IC is classified into ulcerative IC and non-ulcerative IC based on the cystoscopic findings of the presence of Hunner’s lesions and glomerulations that develop after hydrodistention [4,5]. The previous consensus is that IC should be considered if the bladder symptoms persist for more than 6 months, remain refractory to medical therapy, and no other urinary tract diseases are discovered [6]. Since IC is a diagnosis of exclusion, it is rational to exclude any possible etiology for the presenting symptoms of bladder pain, frequency, and urgency.

Controversy exists between the eastern and western concepts of the diagnosis of IC. In the previous National Institute of Diabetes and Digestion and Kidney Diseases (NIDDK) criteria, cystoscopic hydrodistention was recommended to diagnose IC and determine the maximum bladder capacity; however, strict application of the NIDDK criteria would have misdiagnosed more than 60% of patients regarded by researchers as definitely or likely to have interstitial cystitis [6]. The Asian IC guideline followed NIDDK recommendation [7]. However, recent IC guidelines from the Society of Urodynamics and Female Urology do not recommend cystoscopic hydrodistention as a test for the diagnosis of IC [8]. IC should be considered when patients perceive pain, pressure, or discomfort that is related to the bladder with at least one urinary symptom [8]. Cystoscopy is recommended only when it is needed clinically [8]. The main reason is that glomerulation is not specific for the diagnosis of IC because many asymptomatic patients or patients with diseases outside the urinary bladder also develop glomerulations after hydrodistention [9,10].

Another diagnostic test for IC, the potassium chloride (KCl) sensitivity test (PST), which has previously been considered a sensitive indicator of IC, has also lost its role in the diagnosis of IC [11]. Glycosaminoglycan (GAG) is a part of the normal bladder epithelium and protects the bladder mucosa from bacterial adhesion and penetration by toxin substances in the urine [12]. A subset of patients with frequency urgency syndrome has a leaky epithelium and potassium cations diffuse subepithelially and provoke urgency frequency [13]. Intravesical KCl (0.4 M, 40 ml) can elicit abnormal epithelial permeability responses in the diseased bladder, for example, in acute bacterial cystitis, irradiation cystitis, and IC [14]. However, the PST does not correlate with bladder capacity or cystoscopic findings [10]. Interestingly, 88.6% of men with chronic prostatitis also had a positive PST [10].

Research on urothelial dysfunction in IC has recently shown increased inflammation in the suburothelium, decreased urothelial cell proliferation, and increased apoptosis of urothelial cells [15]. Inflammation of the urothelium and suburothelium induces apoptosis through the Bax and BAD pathways [16]. Additionally, the grade of urothelial dysfunction correlated with bladder pain severity and decreased bladder capacity in IC patients [15]. With repeated injections of botulinum toxin A, the inflammation and apoptosis improve, resulting in an improvement of bladder symptoms and capacity [17].

In patients with non-IC lower urinary tract disease (LUTD) such as urinary tract stones, urothelial dysfunction also occurs, resulting in irritative bladder symptoms and glomerulations after cystoscopic hydrodistention [18]. This evidence supports the notion that urothelial dysfunction presents in IC and non-IC LUTD. The increased bladder sensation, frequency urgency, and bladder pain might result from urothelial dysfunction. Therefore, the positive PST and glomerulations developed after hydrodistention, which is consistent with the concept of urothelial inflammation and increased apoptosis of urothelial cells [11]. The bladder dysfunction in these non-ulcer IC and LUTDs may be considered as a broader category of bladder sensory disorder which might be better named ‘hypersensitive’ bladder. In contrast, ulcerative IC has characteristic lesions, which might be regarded as one independent category of bladder pain syndrome [19].

In this study, we revisited the roles of cystoscopic hydrodistention and PST for the diagnosis of IC. We investigated the predictive values of these two old tests in patients with clinically diagnosed IC and other LUTDs. The results of this study provide evidence for the clinical diagnosis of IC.

## Materials and Methods

We prospectively enrolled 214 consecutive patients who had been clinically diagnosed with IC. We also enrolled 125 patients with non-IC LUTD undergoing video urodynamic studies and PST, and another 144 patients with non-IC LUTD undergoing transurethral surgery and cystoscopic hydrodistention. A PST was performed after the video urodynamic studies in all 214 IC patients and the 125 non-IC patients who had video urodynamic studies. Cystoscopic hydrodistention was performed in all 214 IC patients and the 144 non-IC patients who underwent it before transurethral surgery. The LUTDs of the patients undergoing PST and cystoscopic hydrodistention are listed in Table 1.

**Table 1:** Prevalence of positive potassium sensitivity tests (PSTs) in patients with interstitial cystitis (IC) and various non-IC diseases of the lower urinary tract.

	Positive PST- pain	Positive PST- urge	Negative PST
**IC (n = 214)**	150 (70.1%)	33 (15.4%)	31 (14.5%)
Male (n = 30)	20 (66.7%)	4 (13.3%)	6 (20%)
** Female(n = 184)**	130 (70.7%)	29 (15.8%)	25 (13.6%)
**Non-IC (n = 125)**	16 (12.8%)	7(5.6%)	102 (81.6%)
** BOO (n = 25)**	1 (4%)	1 (4%)	23 (92%)
** DO (n = 32)**	5 (15.6%)	2 (6.3%)	25 (78.1%)
** HSB (n = 17)**	5 (29.4%)	2 (11.8%)	10 (58.8%)
** NVD (n = 3)**	0	0	3 (100%)
** SUI (n = 27)**	5 (18.5%)	0	22 (81.5%)
** LUTS (n = 21)**	0	2 (9.5%)	19 (90.5%)

Sensitivity = 183/214 = 85.5%. Specificity = 102/125 = 81.6%. PPV = 183/206 = 88.8%. NPV = 102/133 = 76.7%

BOO: bladder outlet obstruction,

DO: detrusor overactivity,

HSB: hypersensitive bladder,

NVD: neurogenic voiding dysfunction,

SUI: stress urinary incontinence,

LUTS: lower urinary tract symptoms.

This study had been approved by the Institution Review Board of the Buddhist Tzu Chi General Hospital (IRB: 100–06). Written informed consent was obtained from the patients before all procedures, and all patients were informed of the possible adverse events related to the procedures.

Careful history taking, physical examination, symptom assessment, IC questionnaires, and a 3-day voiding diary were conducted in all IC patients on an outpatient basis. Urinalysis, urinary cultures, and urinary cytology were completed in all enrolled patients to exclude urinary tract infections and bladder cancer.

The PST was performed immediately after the video urodynamic studies. After drainage of all post-void residual urine, 40 ml of 0.4-M KCL solution was slowly instilled into the bladder [14]. Patients were requested to report their bladder sensations during KCl solution instillation and compare that sensation with the physiological saline instilled during the video urodynamic studies. If the patient felt bladder discomfort or a painful sensation during PST, they were asked to grade the bladder discomfort on a 10-point visual analogue scale (VAS). Only a bladder pain score of ≥ 3 or a sudden onset of urgency sensation elicited by the PST was considered a positive result. If patients did not feel bladder pain but experienced an urgency sensation during PST, they were asked to grade the severity of the sensation based on the urgency severity score (USS) [20]. A USS ≥ 1 was also considered a positive PST. If patients felt bladder pain with a VAS of < 3 or no urgency, they were regarded as having a negative PST.

After completing the examinations at the outpatient clinics, patients with IC were admitted for a 2-day hospitalization for cystoscopy and hydrodistention under anesthesia. Cystoscopic hydrodistention was performed with the patient under general anesthesia and with an intravesical pressure of 80 cm H_2_O for 15 minutes, and the bladder was evacuated slowly. Formation of petechia, glomerulations, splotch hemorrhages, mucosal fissures, and ulceration was carefully assessed [7]. Cystoscopic hydrodistention was performed by a single surgeon (Kuo HC). Results from the cystoscopy and bladder hydrodistention were recorded. From this data, the maximal anesthetic bladder capacity (MBC) and the grade of glomerulations (0–3) were obtained as described in the Interstitial Cystitis Data Base study [21]. Among the 144 patients who underwent transurethral surgery, cystoscopic hydrodistention was also performed before surgery. The grade of glomerulations and MBC were also recorded.

Continuous variables were expressed as means ± standard deviations (SDs), and categorical data were expressed as numbers and percentages. Statistical comparisons between the groups were conducted using the Fisher exact test for categorical variables and analysis of variance for continuous variables. We also calculated the sensitivity, specificity, positive predictive value (PPV), and negative predictive value (NPV) of the two diagnostic tests. All statistical assessments were two-sided and considered significant at a value of *P* < 0.05. The statistical analyses were performed using SPSS version 15.0 statistical software (SPSS Inc., Chicago, IL, USA).

## Results

A total of 214 patients (30 men and 184 women) with a clinical diagnosis of IC were enrolled. All IC patients underwent video urodynamic studies, PST, and cystoscopic hydrodistention. In addition, 125 patients with non-IC LUTD who were undergoing video urodynamic studies also had a PST (Table 1). Cystoscopic hydrodistention was performed in another 144 patients undergoing transurethral surgery due to bladder outlet obstruction (BOO, n = 32), stress urinary incontinence (SUI, n = 17), upper urinary tract stones (n = 67), idiopathic hematuria (n = 5), and other urinary tract diseases.

Table 1 shows the percentages of positive PSTs among IC and non-IC patients. The PST test for the diagnosis of IC had a sensitivity of 85.5%, specificity of 81.6%, PPV of 88.8%, and an NPV of 76.7%. There was no significant difference of a positive PST between genders.

Table 2 shows the MBC and glomerulation grade in IC and non-IC patients under cystoscopic hydrodistention. Glomerulations developed in 211 (98.6%) IC patients and 61 (42.4%) non-IC patients. No significant difference of MBC and distribution of glomerulation grade between genders. Glomerulations developed in other urinary tract diseases, particularly in patients with stones (67.2%), followed by hematuria (40%) and SUI (35.3%). If we defined positive glomerulation in IC patients as grade 1 or greater, the sensitivity was 98.6%, and the specificity was 57.5%, the PPV was 77.6%, and the NPV was 96.5%. If we defined positive glomerulation as grade 2 or more, the sensitivity was 61.7%, and the specificity was 84.0%, the PPV was 85.2%, and the NPV was 59.6%.

**Table 2:** Maximum bladder capacity and glomerulation grade in interstitial cystitis patients and patients with other urinary tract diseases.

	MBC (ml)	Glomerulations				Positive
		G0	G1	G2	G3	Glomerulations
IC (n = 214)	691.5±242.0	3	79	118	14	211/214 (98.6%)
Male (n = 30)	701.4±215.2	0	12	17	1	30/30 (100%)
Female (n = 184)	689.1±234.7	3	67	101	13	181/184 (98.4%)
Non-IC (n = 144)		83	38	20	3	61/144 (42.4%)
BPO (n = 32)	556.6±203.6	28	3	1	0	4/32 (12.5%)
SUI (n = 17)	747.4±195.2	13	1	5	0	6/17 (35.3%)
Stone (n = 67)	667.2±170.3	22	31	13	1	45/67 (67.2%)
Hematuria (n = 5)	610.0±274.8	3	1	0	1	2/5 (40%)
Others (n = 21)	685.2±212.3	17	2	1	1	4/21 (19.0%)

For G1: Sensitivity = 211/214 = 98.6%. Specificity = 83/144 = 57.5%. PPV = 211/272 = 77.6%. NPV = 83/86 = 96.5%.

For G2: Sensitivity = 132/214 = 61.7%. Specificity = 121/144 = 84.0%. PPV = 132/155 = 85.2%. NPV = 121/203 = 59.6%.

IC: interstitial cystitis,

MBC: maximal bladder capacity,

G: grade,

BPO: benign prostatic obstruction,

SUI: stress urinary incontinence.

Table 3 shows the urodynamic characteristics of IC patients with positive and negative PSTs. IC patients with a positive PST had a significantly smaller urgency sensation capacity, smaller voided volume, and greater bladder pain. However, there was no significant difference in the age, gender or grade of glomerulation after hydrodistention.

**Table 3:** Urodynamic parameters, pain symptoms and glomerulation grades of 214 interstitial cystitis patients with positive or negative potassium sensitivity tests.

	Positive PST (n = 183)	Negative PST (n = 31)	P value
**Sex**			
** Female**	159 (86.4%)	25 (13.6%)	0.574
** Male**	24 (80%)	6 (20%)	
**Age (yeas)**	48.6 ± 14.4	47.2 ± 10.2	0.552
**Urodynamic study**			
** FSF (ml)**	123.9 ± 52.9	116.5 ± 36.3	0.450
** FS (ml)**	193.1 ± 74.1	210.1 ± 61.7	0.229
** US (ml)**	235.9 ± 83.2	280.9 ± 86.4	0.006
** Pdet (cmH2O)**	21.5 ± 12.7	23.4 ± 15.7	0.461
** Qmax (ml/s)**	12.8 ± 5.3	15.2 ± 6.1	0.030
** Void volume(ml)**	254.8 ± 97.0	355.5 ± 155.8	0.002
** PVR (ml)**	22.2 ± 44.5	43.5 ± 42.5	0.415
** CBC (ml)**	282.5 ± 98.6	376.6 ± 178.9	0.007
**Pain**			
** Positive**	140 (76.5%)	2 (6.5%)	0.000
** Negative**	43 (23.5%)	29 (93.5%)	
**Glomerulations**			
** Grade 0**	3 (1.6%)	0	0.395
** Grade 1**	64 (35%)	15 (50%)	
** Grade 2**	104 (56.8%)	14 (46.7%)	
** Grade 3**	12 (6.6%)	1 (3.3%)	

PST: potassium sensitivity test,

FSF: first sensation of filling,

FS: full sensation,

US: urge sensation,

Pdet: detrusor pressure,

Qmax: maximum flow rate,

PVR: post-void residual,

CBC: cystometric bladder capacity.

## Discussion

The results revealed that a positive PST has a good sensitivity (85.5%) and specificity (81.6%) for the diagnosis of IC. Cystoscopic hydrodistention and grade 1 glomerulation is also highly sensitive (98.6%) but less specific (57.5%) for the diagnosis of IC. A positive PST indicates a more hypersensitive bladder with pain symptoms. However, it cannot predict the grade of glomerulation after hydrodistention. Although neither test provided 100% diagnostic accuracy for IC, we might select patients into different subgroups based on different PST and cystoscopic hydrodistention results, not for making a diagnosis of IC but for guidance of different treatments.

Diagnosis of IC is usually based on the clinical symptoms [1]. Hydrodistention under general anesthesia has been considered the classic diagnostic test to demonstrate glomerations and mucosal fissures in IC but has now been largely abandoned [8]. A positive PST provides a diagnosis of urothelial leakage and a measurement of the therapeutic effect of protective surface treatment [11]. In a recent clinical study, a positive PST causing bladder pain of more than 2 on the VAS and small cystometric capacity were 100% predictive of IC in patients with hypersensitive bladder syndrome [22].

The urothelium plays a significant role in communicating with bladder nerves, smooth muscle, and the inflammatory system and cells [23]. A leaky epithelium is considered the primary disease that causes urinary potassium leakage into the suburothelium and generates symptoms of frequency urgency and bladder pain [24]. Intravesical KCl instillation at a concentration of 0.4 M provoked symptoms in patients with IC and other LUTDs [24]. Intravesical sulfated polysaccharide restored injured urothelium to normal [25]. A recent study demonstrated that intravesical GAG replenishment therapy also produced a physiological decrease in the recruitment of inflammatory cells in a rat model of acute bladder damage [26].

Recent studies have shown increased apoptosis of the urothelium in patients with IC [16]. Terminal deoxynucleotidyl transferase dUTP nick end labeling (TUNEL) staining showed apoptotic cells in the microvascular endothelial cells in IC/bladder pain syndrome (BPS) bladders [27]. Higher urothelial cell apoptosis and abnormally lower E-cadherin expression in IC bladders are associated with chronic inflammation [16]. This evidence reveals that a positive PST indicates an inflamed urothelium in IC bladders; however, in some types of LUTDs, the bladder urothelium might also be damaged, and therefore, these patients might also have a positive PST [10, 24]. Once the underlying pathology is resolved, the urothelial dysfunction often disappears.

Glomerulation during cystoscopic hydrodistention is highly associated with overexpression of angiogenic growth factors such as platelet derived endothelial cell growth factor or thymidine phosphorylase [28], vascular endothelial growth factor (VEGF) [29], and hypoxia-inducible factor-1-alpha [30]. VEGF, which plays a critical role in bladder inflammation, is closely associated with the vascular alterations observed in patients with IC/BPS. Increased VEGF was associated with bladder inflammation and a smaller functional bladder capacity in IC patients. Interestingly, we also found that glomerulation after cystoscopic hydrodistention is frequently encountered in patients with upper urinary tract stones [18]. This phenomenon reflects that cross talk occurs between the upper and lower urinary tracts. A stone in the upper urinary tract might induce an inflammatory reaction, which may affect the urinary bladder and on some occasions, result in irritative bladder symptoms. In the differential diagnosis of IC, urinary tract stones should be placed at a high priority.

In this study, we found that grade 1 glomerulation had a sensitivity of 98.6% for IC, but the specificity was only 57.5%. When we increased the glomerulation to grade 2, the sensitivity decreased to 61.7% while the specificity increased to 84% for IC. Grade 2 glomerulation involves at least 3 or more quadrants of the bladder wall with glomerulations after cystoscopic hydrodistention, indicating a higher grade of inflammation in the bladder wall. Although the incidence of grade 2 glomerulation with urinary tract stones remains high, after exclusion of urinary tract stones, we detected patients who had urothelial dysfunction using this test.

As shown in Table 3, a positive PST in IC patients was significantly associated with a small bladder capacity triggering urgency sensation, small voided volume, and cystometric bladder capacity, indicating that bladder hypersensitivity is associated with a leaky urothelium. Furthermore, 76.5% of IC patients with a positive PST had bladder pain whereas bladder pain occurred in only 6.5% of patients with a negative PST test, suggesting that a leaky urothelium might be the source of bladder pain in IC patients. However, because the grade of glomerulation after hydrodistention was not associated with the PST results, a leaky urothelium and glomerulations might be an independent pathophysiology of IC.

Increased urothelial permeability could be a cause and also a result of LUTD. Urothelial dysfunction might be a more appropriate term to describe this pathophysiology, which may be found not only in IC but also in recurrent UTI, urinary tract stones, and chronic prostatitis. Therefore, urothelial dysfunction is not a pathognomonic disorder of IC; it is also be found in patients with BOO, DO, HSB, or SUI when these patients have chronic inflammation in the bladder wall. In patients with LUTS and a positive PST, we should first search for some correctable etiologies before making a diagnosis of IC. In this regard, a video urodynamic study is important to be able to exclude BOO in patients with IC symptoms.

The strength of this study is that we enrolled many different non-IC patients for PST and cystoscopic hydrodistention. The results support that we could not make a diagnosis of IC solely based on a positive PST or glamerulation after cystoscopic hydrodistention. The limitation is that only clinically diagnosed IC patients, but not all patients with hypersensitive bladder and bladder pain patients, were enrolled. In addition, we did not enroll completely LUTS free subjects as the controls. The PST was performed after urodynamic test which might result in minor urothelial injury during catheterization and causing false positive PST.

## Conclusions

The study concluded that both a positive PST and glomerulation after hydrodistention are sensitive indicators of IC. The specificity of glomerulation is lower than that of PST. A positive PST is associated with a hypersensitive bladder and bladder pain in IC patients. However, IC is a diagnosis made on clinical grounds and neither test allows one to definitely make a diagnosis. The results of PST and cystoscopic hydrodistention do not render a diagnosis of IC, but might provide a guide of different treatments. In the diagnosis and treatment of IC, any potential urinary tract diseases should be carefully evaluated and excluded.
